# A Zero-Brine Discharge Seawater Desalination Using a Pilot-Scale Membrane Distillation System Integrated with Crystallizer

**DOI:** 10.3390/membranes12080799

**Published:** 2022-08-19

**Authors:** Jian Zuo, Chin Ann Chow, Ludovic F. Dumée, Antony J. Prince

**Affiliations:** 1 Food Chemical and Biotechnology, Singapore Institute of Technology, 10 Dover Drive, Singapore 138683, Singapore; 2 Department of Chemical Engineering, Khalifa University, Abu Dhabi P.O. Box 127788, United Arab Emirates; 3 Center for Membranes and Advanced Water Treatment (CMAT), Khalifa University, Abu Dhabi P.O. Box 127788, United Arab Emirates; 4 Memsift Innovations Pte Ltd., 192 Pandan Loop, Singapore 128381, Singapore

**Keywords:** reverse osmosis, brine management, membrane distillation, zero liquid discharge, water recovery, selective crystallization

## Abstract

The management of brines generated from reverse osmosis operation remains a critical challenge requiring new approaches and processes to limit the impact of brine discharge onto ecosystems and to enhance both water and valuable resource recovery. The treatment of real seawater reverse osmosis (SWRO) brines (45,000 ppm TDS) obtained from a local Singaporean desalination plant with a crystallizer integrated pilot-scale membrane distillation unit (MDC) was studied. Commercial STOMATE^®^ hollow fiber membranes were used in vacuum membrane distillation (VMD) configuration, leading to an average flux of around 3.7 L/m^2^-h at a permeate vacuum of 80 kPa and an average feed temperature of 65 °C. Consistent separation operations were achieved for the treatment of real SWRO brine over a period of 280 h; this led to a water recovery of >95% and to the collection of salt slurries, containing up to ~10–20 wt% of moisture, from the crystallizer. This approach demonstrates the potential of MDC systems to achieve zero brine discharge efficiently from seawater desalination systems, providing an environmentally friendly alternative to manage brines by increasing water recovery and generating salt slurries of economic value.

## 1. Introduction

Although nearly 75% of Earth’s surface is covered with water, over 97% is saline with only ~2.5% available as freshwater for human activities [1]. Out of this 2.5%, a fraction is, in fact, readily reachable; moreover, increasing global shortages of freshwater are aggravated by climate change, widespread urbanization and persistent pollution of freshwater sources [2,3,4]. Technologies to provide fresh and potable water for daily activities, such as irrigation, animal and human consumption, are therefore critical; various conventional desalination technologies have been established commercially to generate freshwater from seawater [3].

The techno-economical drawbacks of conventional desalination technologies, such as distillation systems and reverse osmosis (RO), include large energy penalties; these result in consequent operating costs [5,6]. Distillation systems typically operate at temperatures close to the boiling point of water; this also increases the risk of scale formation within the heat exchangers and of corrosion of the systems [6]. The footprint of RO systems, the current leading technology for seawater desalination, is also relatively large; in addition, pre-treatment steps must be carried out to ensure the removal of the majority of potential foulants. This would otherwise lead, over time, to the deterioration and clogging of membranes; in turn, this would require extensive operational costs towards cleaning and regeneration operations [7,8].

RO operations also lead to the production of brines, which must be managed and discarded; thus, this represents a critical environmental challenge to exposed ecosystems [9,10,11]. RO brines are very saline, at least twice more concentrated than seawater; in addition, they may contain various dissolved solids, including silica or metal salts [12]. Brines may also contain chemicals, added during the various pre-treatment stages of RO, such as flocculants, coagulants and anti-scalants, as well as biological contaminants imported from the original seawater sourcing [12]. Sensitive marine species may be harmed by subtle changes in salinity, as low as 2–3 parts per thousand, to pH variations and to the presence of toxic dissolved solids occurring within RO brines [13]. The toxicity of chemicals and ions present within the brines will negatively impact marine ecosystems and natural balance [12]. Appropriate brine management is, therefore, a major challenge to be urgently tackled.

RO brines are currently disposed either as surface water discharges or sewer discharge during deep-well injection or concentrated in evaporation ponds [12]. In the United States, 27% of the plants use sewage disposal [14]; this leads to control challenges to meet sewer quality standards prior to the final disposal of the water as surface water discharge at sea. The potential use of brine doped sewage waters for irrigation will also be affected due to the increased levels of total dissolved solids (TDS); this makes these effluents unfit for direct agricultural applications [14,15]. Both deep-well and evaporation ponds have less restrictions on the quality of the discharged waters; however, they require appropriate land management and represent significant capital costs. Furthermore, the possibility of leakages often discourages the use of such structures [12]; more effective strategies towards the reduction and management of RO brines must be developed.

Alternative technologies for RO brines treatment include forward osmosis (FO), multistage evaporative distillation (MED), multistage flash distillation (MSF) as well as membrane distillation (MD) coupled with membrane crystallization (MC) [16,17]. MD is a promising technology due to operating at low operation pressures and temperatures, when compared to other conventional membrane and thermal methods; moreover, it offers a 100% theoretical rejection to salts. MD may also be operated on low-grade heat sources or based upon waste-heat collection systems; in addition, it typically offers a reasonably smaller footprint than conventional desalination systems. Last but not least, the MD process is nearly insensitive to the feed salinity for desalination; this makes it invaluable for high-salinity effluents management [18,19,20,21,22,23,24,25,26,27,28,29,30]. The challenges of MD, however, include membrane scaling and long-term membrane pore wetting; these require the development of advanced materials and flow dynamics.

The development of efficient MD membrane materials remains a major bottleneck towards successful MD process scale-up. The hydrophobicity and surface energy of the surface of the pores of the membranes, as well as the pore size distribution and thickness of the active layer involved in the separation, will dictate the long-term performance and the stability of the operation [25] to prevent wetting and be able to last for a long period of time [31]. The engineering of appropriate membrane materials is of particular concern when treating high-salinity solutions or high chemical concentrations such as RO brines; this is due to the greater risks of membrane fouling or scaling and deterioration [32]. Direct contact membrane distillation (DCMD) setups involving poly(vinylidene fluoride) (PVDF) membranes were developed to treat simulated RO brine. Although this approach led to a water recovery of over 70%, significant scaling occurred upon reaching the concentration factors: defined as 1/(1-water recovery ratio), beyond 3.3. This scaling resulted in a drop in thermal performance and serious pore wetting [33], pointing out at the need for novel membrane materials development.

In this study, the potential of a commercial hollow fiber membrane, STOMATE^®^ membrane from Memsift Innovations Pte Ltd., was evaluated for the concentration of seawater reverse osmosis (SWRO) brines. The physicochemical properties, as well as the long-term chemical stability and mechanical properties of the membranes, were firstly characterized; this occurred prior to evaluating the MD performance with synthetic seawater solutions and a pilot-scale MD system with SWRO brines collected from a local desalination plant in Singapore. The long-term performance, thermal efficiency, scaling tendency and cleanability of the membranes were investigated during the pilot study to push the limits of these novel membranes; this was conducted so as to concentrate the SWRO brines to near saturation point to collect the salt crystals separately. This approach offers, for the first time, a unique perspective on the scalability of MD to support large- scale brine treatment and operation; it also provides the opportunity to have a better understanding of membrane scaling during extreme salinity operation.

## 2. Experimental

### 2.1. Materials

Hollow fiber membranes, STOMATE^®^, were provided by Memsift Innovations Pte Ltd from Singapore. The membrane specifications are presented in Table 1. Sodium chloride (NaCl) was purchased from Sigma Aldrich (Singapore). Sodium hydroxide (NaOH) and hydrochloric acid (HCl) were purchased from Merck (Singapore). Deionized (DI) water was produced by the Sartorius water purification system. STOMATE^®^ membranes are composite hydrophobic membranes containing proprietary additives, yielding extremely relevant properties for MD [34,35].

For the pilot operations, the STOMATE^®^ membranes were assembled into a pilot-scale testing module with an effective membrane area of 0.2 m^2^. The modules were supplied by Memsift Innovations Pte Ltd. Real SWRO brine (45,000 ppm TDS) was collected from a local seawater desalination plant in Singapore.

### 2.2. Membrane Characterization Techniques

#### 2.2.1. Scanning Electron Microscopy (SEM)

Scanning electron microscopy (JEOL JSM-IT300, Tokyo, Japan) was used to observe the surface and cross-section morphologies of hollow fiber membranes. The SEM samples were prepared by using liquid nitrogen to freeze fracture the membranes; and then being coated with a thin conductive platinum layer using a JEOL JEC-3000FC ion sputtering device. The images were acquired under an accelerating voltage of 10 KV and a working distance of 15–16 mm.

#### 2.2.2. Tensile Strength

The mechanical properties of the hollow fibers were measured with a tensiometer (MTS Criterion Series 40 C41.103–100N, Minnesota, USA). In order to evaluate the long-term mechanical stability of the membranes, the samples were immersed in different pH solutions of 1, 3, 7 and 10 for a duration of 10 weeks. The testing of the tensile properties was performed on a weekly basis and the changes in tensile strength were recorded. For all the measurements, the starting gauge length was 50 mm and the elongation rate was 10 mm/min.

#### 2.2.3. Morphological Stability Tests

Morphological stability tests of the membranes were performed over the course of 10 weeks. The membranes were submerged into solutions at a pH of 1, 3, 7 and 10 to assess any morphological changes in length and weight. The dry weight and length of membrane samples were firstly recorded by using a balance and Vernier calipers, respectively. After being immersed for 20 days, the first measurement was made. Subsequently, measurements were performed daily until week 10. Prior to each measurement, the membranes were taken out from the solutions and dried in a vacuum oven (Binder VDL 53, Tuttlingen, Germany) at 30 °C.

#### 2.2.4. Porosity Test

The porosity of the membranes is described as the volume of the pores divided by the total volume of the membranes, which is the void volume fraction of the membrane [36]. The Equation (1) shows the calculation of porosity, ε [37]:

(1) $$\varepsilon =\frac{\left(m_{f}-m_{i}\right)}{\rho_{H2O}\times V_{i}}\times 100$$

where $m_{i}$ (kg) is the initial dry mass of the fiber and $m_{f}$ (kg) is the final mass of the fiber after being soaked in water; the excess water on the surface was wiped away and the fiber was weighed again to determine $m_{f}$. $V_{i}$ stands for volume of the fiber in m^3^ and $\rho_{H2O}$ is 1000 kg/m^3^.

### 2.3. Membrane Distillation

Lab-scale experiments were conducted with a small lab module assembled from 4 hollow fiber membranes under the DCMD mode, which had an effective membrane area of 20 cm^2^. Solutions composed of 3.5, 10 and 15 wt% NaCl were used as feed solutions to perform the experiments at 50, 60, 70 and 80 °C, respectively. DI water was used as the permeate solution, wherein the temperature was controlled at 15 °C. The feed solution was circulated through the lumen side of the hollow fiber, while DI water was circulated across the shell side using peristaltic pumps (Masterflex L/S). Both the feed and permeate flow rate was kept at 0.1 L/min. The Reynolds number (Re) can be calculated using the formula:

$$Re=\frac{\rho VD}{µ}$$

where ρ is solution density (kg/m^3^), *V* is the flow velocity (m/s), *D* is the diameter of the fiber (m) and µ is the viscosity (N.s/m^2^). The *Re* value of the feed was estimated to be 1030. This is still in the laminar flow region.

The conductivity and weight changes of the permeate solution were closely monitored with a conductivity meter and a balance (Mettler Toledo). Measurements were made at 10 min intervals and salt rejection was calculated using Equation (2) [36,37]:

(2) $$\beta =\left(1-\frac{C_{p}}{C_{f}}\right)\times 100\%$$

where $C_{p}$ and $C_{f}$ are the NaCl concentrations in permeate and feed solutions, respectively. The permeate concentration was calculated from Equation (3) based on the dilution effect:

(3) $$C_{p}=\frac{C_{1}m_{1}-C_{0}m_{0}}{m_{1}-m_{0}}$$

where $m_{1}$ and $m_{0}$ are the final and initial masses of permeate. $C_{1}$ and $C_{0}$ are the final and initial salt concentrations of permeate, which could be determined from the conductivity.

The flux was calculated by using the sample weight collected at 10 min intervals divided by the area of membrane and converted to L/m^2^-h [36,37]:

(4) $$Flux=\frac{m}{A\times t\;}$$

where m is the mass (kg) of the permeate sample collected over time t (h) and *A* is the effective membrane area in m^2^.

**Figure 1:**
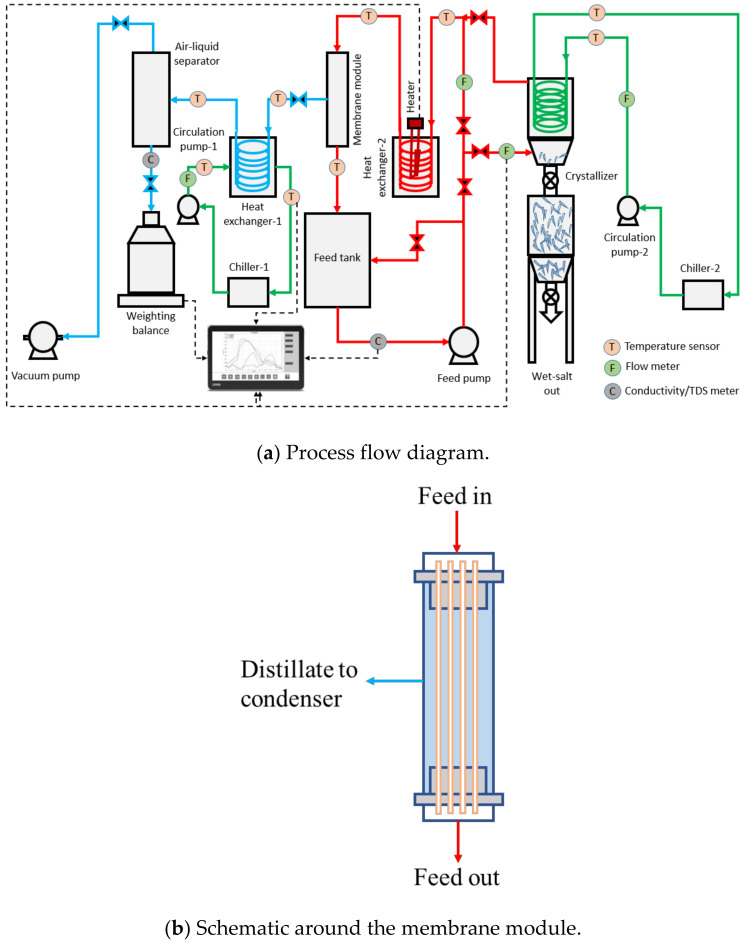

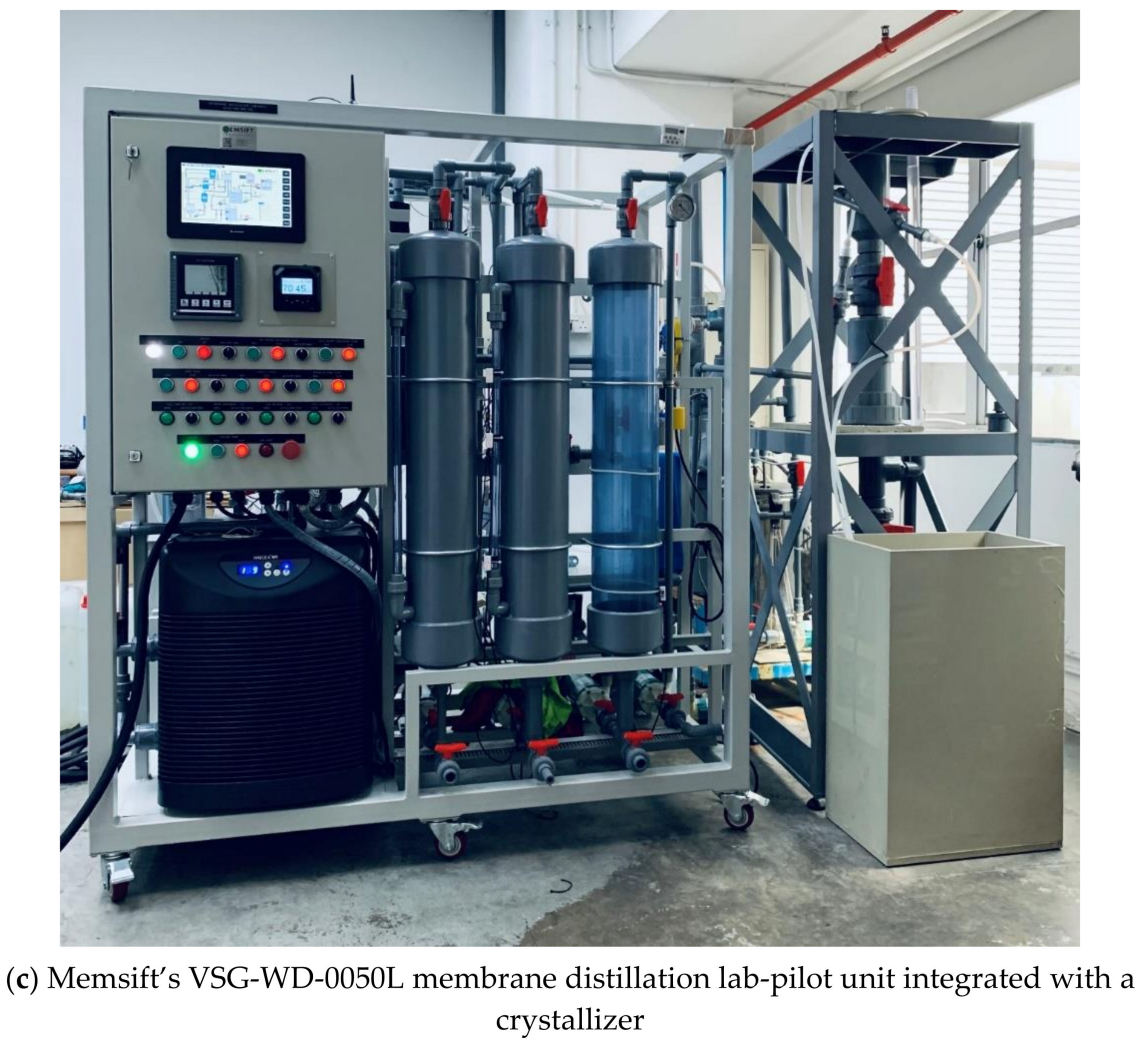
(**a**) Process flow diagram, (**b**) schematic around the membrane module and (**c**) actual image of the zero liquid discharge system.

During the pilot-scale studies, the real SWRO brine collected from a local desalination plant in Singapore was used as the feed solution using Memsift’s VSG-WD-0050L membrane distillation lab-pilot unit; this was further integrated with a salt separator/crystallizer. Figure 1a shows the process flow diagram of the entire zero liquid discharge system; Figure 1b shows the flow schematics around the membrane module; and Figure 1c shows the actual image of the system. The RO brine was heated to 65 ± 2 °C using an electrical heater; the hot feed was then pumped into the lumen side of the membrane module with a velocity of 0.42 m/s, which gave a Re number of 1376. The water vapor was drawn upon application of vacuum, at a pressure of 80 kPa on the permeate side upstream from a condenser operated with cooling water at a temperature of 27 ± 3 °C; the condensed permeate was collected in the permeate collection container. The concentrated brine was pumped to a crystallizer at a temperature of 13 ± 2 °C and at a flow rate of 0.2 L/min; here, the saturated brine was crystalized and separated by gravity with minimal circulation while the supernatant was heated up and recirculated back into the MD system. The TDS of the permeate was measured continuously to determine the efficiency of the membrane separation process, and the calculation of salt rejection and flux were performed. The experiment was run during the daytime, 8 h/day, and stopped overnight for safety reasons. The membrane module was taken out from the system and rinsed with tap water (TDS < 500 ppm) for about 10 min every day after the experiment to prevent surface crystallization and let the membrane dry overnight. The experiments were continued for a period of around ~280 h to fully evaluate the long-term performance of the system.

## 3. Results and Discussion

In this section, the physicochemical properties of the membranes are firstly discussed. Then, the MD performance of the membranes is systematically investigated, using synthetic seawater with various salt concentrations. With the understanding of membrane stability and MD performance, the membranes are tested in a pilot-scale MD system with real SWRO brine. Furthermore, the SWRO brine is concentrated to near saturation; in addition, a crystallizer is coupled with the pilot system to separate the formed salt crystals. This demonstrates the potential of MDC systems to achieve zero brine discharge and to recover valuable resources from the brine.

### 3.1. Membrane Characterization

#### 3.1.1. Morphology of the Membrane Materials

**Figure 2:**
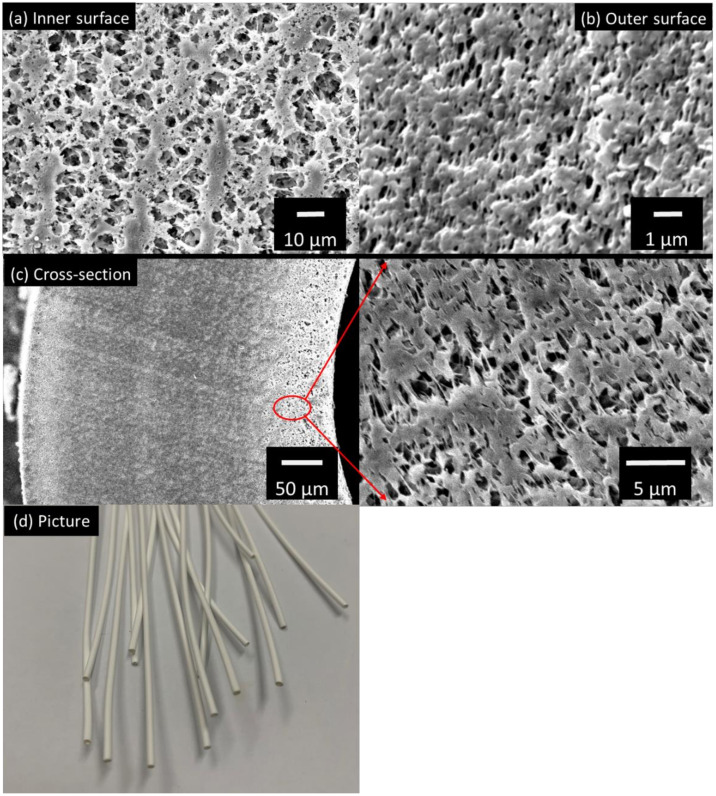
The SEM images of (**a**) the inner surface, (**b**) outer surface, (**c**) cross-section and (**d**) picture of the STOMATE^®^ membrane.

Figure 2a,b show the surface images of the STOMATE^®^ hollow fiber membrane. Both the inner and outer surfaces have macroporous structures with visible pores observed. These macroporous morphologies are desirable for MD applications as they promote vapor transportation. The mean pore size data is provided by the company, which is 0.08 ± 0.02 µm. This value is at the lower end of the typical pore size ranges (0.1–0.6 µm) recommended for MD processes [36,38]. The smaller pore size would help to prevent membrane wetting under different operation conditions, especially when there is a high salt concentration such as in RO brine [39]. Figure 2c displays the cross-section morphology and Figure 2d shows a photographic image of the fiber. The membrane has a porous cross-section and is macrovoid-free. This macrovoid-free structure could help to increase membrane wetting resistance and membrane mechanical properties as compared to macrovoid structures. Moreover, this structure may provide better performance stability during long-term operations. Despite the macrovoid-free structure, the hollow fiber still possesses a good porosity of 65.5%; this is suitable for MD applications [36].

#### 3.1.2. Stability of the Membrane Materials

**Figure 3:**
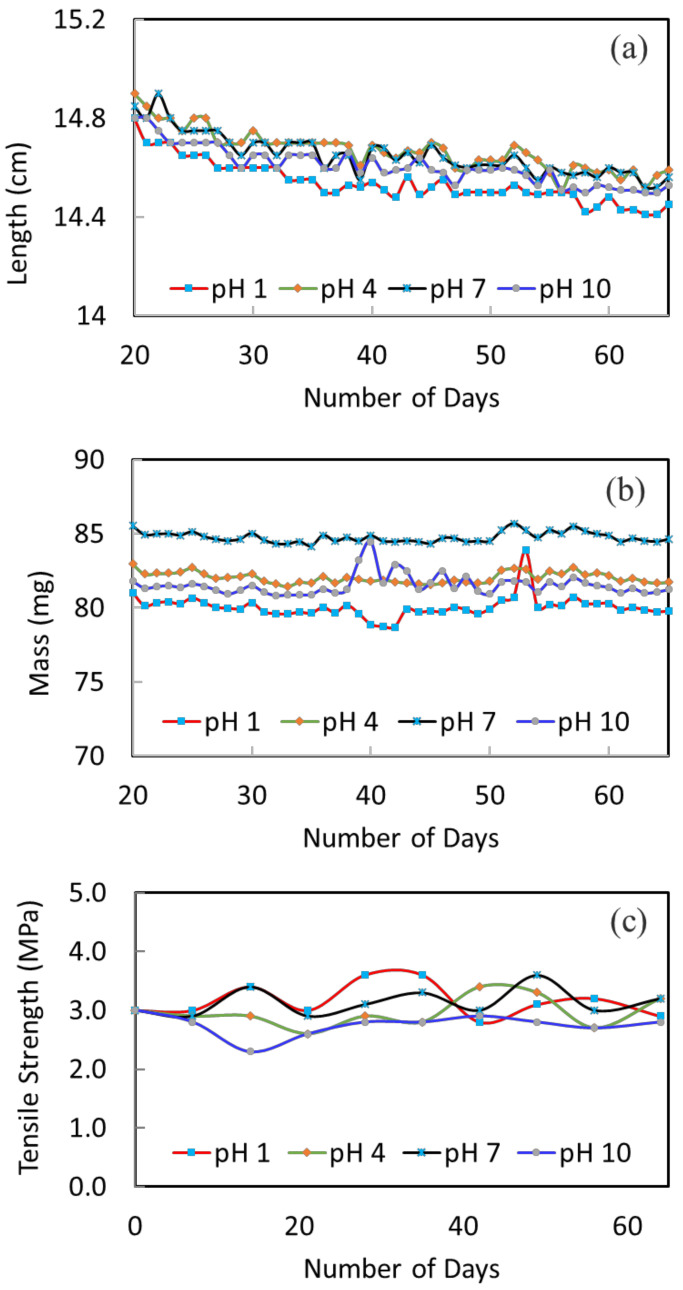
The variation of fiber (**a**) length, (**b**) mass and (**c**) tensile strength after been immersed in the different pH solutions.

The stability of the membranes at different pH conditions was tested over a course of 10 weeks. Figure 3a shows the change of fiber length versus immersion time. The fluctuation of all the curves is due to measurement errors; yet, the overall trend on the variation of fiber length can be clearly seen. Generally, there is a slight decreasing trend (1–2% decrease) until about 50 days; then, the fiber length remains quite constant. The slight decrease of fiber length is because of pore shrinkage and polymer relaxation, which may also be referred to as membrane aging [40,41]. Similar observations were made by Ravereau [40], where the pore size and length of their membranes tended to undergo shrinkages when being submerged in different pH solutions [40]. This is a common phenomenon for polymeric membranes. Aging happens to dry membranes as well as wet membranes. When it happens, the membrane pore size gets smaller and membrane flux will decrease. Therefore, the degree of change in fiber length becomes important. The larger the change, the larger the effect on membrane performance and stability. For the current membranes, the changes are all small. For example, the change in fiber length is about 1.3% for the pH 1 condition; the change is about 1.4% for the pH 10 condition. The smaller variation under different pH conditions indicates a good stability of the membranes. In addition, the fiber length remains constant after about 50 days; this shows that the membranes are stabilized and a stable membrane performance during long-term operation can be expected.

Figure 3b displays the change in fiber weight as a function of immersion time. The mass of fibers under pH 1, 4, 7 and 10 conditions are quite constant, which indicates the good stability of membranes under these conditions.

#### 3.1.3. Mechanical Properties

Figure 3c presents the 10-weeks’ variation of tensile strength to further investigate the mechanical stability of the membrane in different pH solutions. The original hollow fiber shows a good tensile strength of 3.0 MPa. This trend is owing to its macrovoid-free structure. After being immersed in different pH solutions, the tensile strength is relatively stable throughout. After 10 weeks, the tensile strength remains in the range of 2.8 to 3.2 MPa. This further confirms the structural stability of the membrane materials and their applicability to the MD process.

### 3.2. Lab-Scale Membrane Distillation

**Figure 4:**
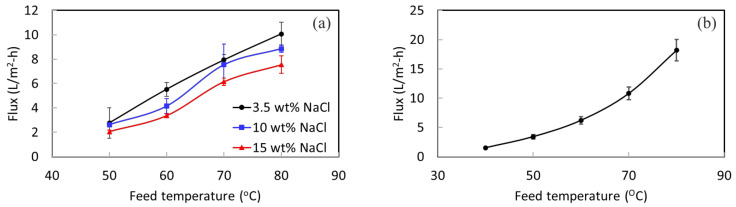
(**a**) DCMD flux under different feed temperatures and concentrations, and (**b**) VMD flux at different temperatures with a 3.5 wt% NaCl feed.

A lab DCMD system was used to perform desalination testing for STOMATE^®^ membranes to understand their basic performance properties. A high rejection value of more than 99.9% is achieved for all the testing. Figure 4 shows the permeate fluxes at different temperatures with different NaCl concentrations. As expected, when the temperature gets higher, the MD flux increases as well. This is because of the increased vapor pressure at a higher feed temperature. The concentration of NaCl increases, while the permeate flux decreases slightly. This is due to the reduction of vapor pressure of the feed under a higher salt concentration [42]. At a feed temperature of 80 °C, a flux of 10 L/m^2^-h is obtained for 3.5 wt% NaCl; 8.8 L/m^2^-h is obtained for 10 wt% NaCl. These results demonstrate that the membrane is capable of treating solutions with high salt concentrations under different operation conditions.

### 3.3. Pilot-Scale Membrane Distillation

The 10-week stability testing showed that the membranes offer long-term structural and mechanical stability. The lab-scale MD experiment indicated that the membrane could withstand different salt concentrations. Therefore, it displayed great potential for pilot-scale testing using real SWRO brine for an extended duration. The experiments with a pilot-scale setup were carried out using vacuum membrane distillation (VMD) with a partial permeate vacuum of 80 kPa. Figure 4b shows the VMD flux of the membrane at different feed temperatures with a 3.5 wt% NaCl feed concentration. Acceptable permeate fluxes are obtained at this low permeate vacuum of 80 kPa. The membrane was then evaluated in the pilot system for its long-term performance.

**Figure 5:**
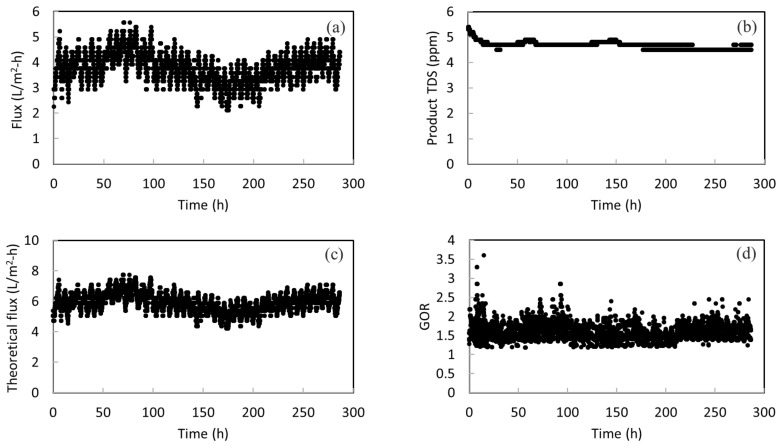
(**a**) The permeate flux for the pilot-scale testing; (**b**) the total dissolved solids (TDS) amount in the product water; (**c**) the theoretical flux calculated for the pilot-scale testing; and (**d**) the GOR of the system with heat recovery from the condenser.

Figure 5a presents the long-term performance of the membrane in treating actual SWRO brine for a period of about ~280 h. The daily fluctuation of membrane flux can be seen. This is probably due to the surface fouling of membranes during continuous operation. Fouling occurs when there are undesired materials on the membrane surface by the adsorption or deposition of foulants [43]. At the end of each day, simple rinsing of the membrane was performed to prevent fouling accumulation and surface crystallization. Then, a higher flux was obtained at the start of the next day. Overall, there is neither large fluctuation nor a sudden change in the membrane flux; thus, this indicates the stability of the membrane in concentrating RO brine. An average flux of 3.7 L/m^2^-h is obtained. It is worth noting that membrane wetting did not happen during the entire operation period. As shown in Figure 5b, the TDS amount in product water remains at around 4.5 ppm. This low TDS value implies a high salt rejection throughout the long-term operation and no membrane wetting occurred. The product water met the salinity standard (500 ppm) of drinkable water set by the World Health Organization (WHO). It shows that more fresh water can be drawn from RO brine with the MD system.

### 3.4. Modelling Performance Data

The theoretical flux of the pilot testing is calculated based on the inlet and outlet temperatures of the module. It assumes 100% energy efficiency where the temperature drop of the feed solution is due to water evaporation. This helps to elucidate the thermal efficiency of the pilot-scale MD system. The theoretical flux can be calculated based on Equation (5) below:

(5) $$J=\frac{Q}{A_{system}}=\frac{mc_{p}\Delta T}{\Delta H_{vaporization}}/A_{system}$$

where J is flux (L/m^2^-h), Q (L/h) is the permeate flow, m is the mass flow rate (kg/s), *c_p_* is the heat capacity (kJ/kg·°C), ΔT is the temperature difference between the feed inlet and outlet (°C), $\Delta H_{vaporization}$ is the latent heat of vaporization, and $A_{system}$ (m^2^) is the surface area of the membrane system.

Figure 5c presents the calculated theoretical flux versus operation time. The theoretical flux is always higher than the actual flux. This is anticipated because the system cannot achieve 100% energy efficiency. There is heat loss to the surroundings and conductive heat loss. The energy efficiency can be simply calculated as the ratio of actual flux over the theoretical flux. By presenting the theoretical flux, the energy efficiency as well as its change over time can be seen clearly. It can be calculated that the energy efficiency is in the range of 55–70% over the course of operation, with an average of 62%. This is within the typical energy efficiency values for systems without heat recovery. According to Fane, due to transmembrane conduction of heat, roughly 20–50% of heat can be lost for their MD processes [44]. According to another study conducted by Bui, where simulation work for MD was performed using a wide range of operating conditions, the highest energy efficiency achieved was 49.9% [45]. Another noteworthy point is that when the actual flux reduces, the energy efficiency also drops. This reveals the importance of membrane stability again. If the membrane does not have a stable performance, the energy efficiency of the system is also sacrificed. Therefore, the stability of the STOMATE^®^ membrane makes it suitable for practical applications.

In addition, the energy efficiency of the system can be further improved by reducing the heat loss or integrating heat recovery designs in the actual process. For example, if the heat is recovered from vapor condensation, the required energy input can be largely reduced and the energy efficiency can be improved. As a demonstration, the gain output ration (GOR) is estimated by subtracting the heat recovered from the condenser from the original heat input to the system. Figure 5d shows that an average GOR of 1.59 can be achieved with heat recovery designs.

### 3.5. Crystallization Operations

**Figure 6:**
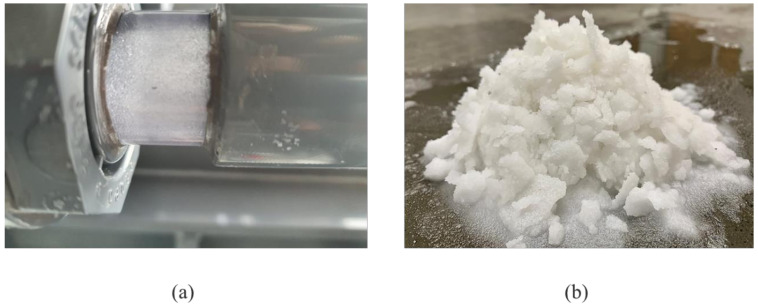
(**a**) Formation of salt crystals in the crystallizer, and (**b**) sea salt recovered from the crystallizer.

During the long-term operation, the salt concentration of the RO brine increased from 4.5% to near saturation point. The temperature of the near-saturated brine was reduced by using the chiller to form solid crystals and the solid crystals were separated in the crystallizer by the gravity deposition method. Figure 6a shows the saturated salt crystals on the side view glass of the crystallizer.

Figure 6b shows the sea salt recovered from the crystallizer. The recovered salt has a moisture content range from 10–20%. The recovered salts could be treated and sold or used for other purposes. Even though SWRO brine is highly saline, the MD membrane copes well in terms of performance. During the entire course of pilot operation, about 19 kg of wet-salt was collected from around 400 L of SWRO brine. The MDC system can continuously remove salt from SWRO brine to generate revenue as well as promote water recovery of up to 99%, considering the minor water lost as moisture in the salt crystals. This provides a more environmentally friendly alternative to RO brine disposal with the advantage of salt recovery and possible zero liquid discharge.

## 4. Conclusions and Prospects

In this study, novel STOMATE^®^ hollow fiber membranes were employed to treat actual SWRO brine at a pilot scale. The physicochemical properties, long-term structural and mechanical stability, as well as the MD performance of the membranes, were investigated. The long-term performance, thermal efficiency, scaling tendency and cleanability of the membranes were explored during the pilot study to push the limits of these novel membranes in order to concentrate SWRO brine to near saturation point to collect salt crystals separately. The following conclusions can be drawn from this study: STOMATE^®^ membranes exhibit macrovoid-free structures with macroporous inner and outer surfaces. They possess an appropriate pore size of 0.08 ± 0.02 µm, porosity of 65.5% and a high wetting resistance. These membranes also show good mechanical properties and stability in acid and alkaline environments up to pH 10. These membranes display good stability in synthetic seawater solutions with various salt concentrations. They also have a stable long-term performance in treating actual SWRO brine under a pilot-scale MD system. A simple washing protocol is developed to rinse the membrane module, which successfully maintains the membrane performance during long-term operation. This demonstrates the feasibility of the MD membrane in actual application and addresses the potential operational issues. A crystallizer is coupled with the pilot-scale MD system to crystallize and separate salt crystals. Salt crystals are successfully recovered in the system. This pushes the limit of the MD system to possibly achieve zero liquid discharge for RO brine, as well as to recover valuable resources from the brine.

## Figures and Tables

**Table 1:** Membrane specifications of the STOMMATE^®^ hollow fiber.

Parameter	Value	Unit
Inner diameter	0.9	mm
Outer diameter	1.6	mm
Thickness	0.35	mm
Bubble point	~0.2	MPa
Pore size	0.08 ± 0.02	µm
Porosity	70 ± 5	%
Hydrostatic pressure	>0.40	MPa

## Data Availability

Data is contained within the article.
